# The effect of chess on cognition: a graph theory study on cognitive data

**DOI:** 10.3389/fpsyg.2024.1407583

**Published:** 2024-09-17

**Authors:** Lissett Gonzalez-Burgos, Candida Lozano-Rodriguez, Yaiza Molina, Eloy Garcia-Cabello, Ramón Aciego, José Barroso, Daniel Ferreira

**Affiliations:** ^1^Departamento de Psicología Clínica, Psicobiología y Metodología, Facultad de Psicología y Logopedia, Universidad de La Laguna (ULL), San Cristóbal de La Laguna, Spain; ^2^Departamento de Psicología Evolutiva y de la Educación, Facultad de Psicología y Logopedia, Universidad de La Laguna (ULL), San Cristóbal de La Laguna, Spain; ^3^Facultad de Ciencias de la Salud, Universidad Fernando Pessoa Canarias, Las Palmas, Spain; ^4^Division of Clinical Geriatrics, Center for Alzheimer Research, Department of Neurobiology, Care Sciences, and Society, Karolinska Institutet, Stockholm, Sweden

**Keywords:** chess, neuropsychology, graph theory, connectome, modularity, efficiency

## Abstract

**Objectives:**

We aimed to advance our understanding of the effect of chess on cognition by expanding previous univariate studies with the use of graph theory on cognitive data. Specifically, we investigated the cognitive connectome of adult chess players.

**Method:**

We included 19 chess players and 19 controls with ages between 39 and 69 years. Univariate analysis and graph theory included 27 cognitive measures representing multiple cognitive domains and subdomains. Graph analysis included global and nodal measures of integration, segregation, and centrality. We also performed an analysis of community structures to gain an additional understanding of the cognitive architecture of chess players.

**Results:**

The analysis of global graph measures showed that chess players had a higher local efficiency than controls at the cost of a lower global efficiency, which did not permeate segregation aspects of their connectome. The nodal graph measures showed that executive/attention/processing speed and visuoconstructive nodes had a central role in the connectome of chess players. The analysis of communities showed that chess players had a slightly reorganized cognitive architecture into three modules. These graph theory findings were in the context of better cognitive performance in chess players than controls in visuospatial abilities.

**Conclusion:**

We conclude that the cognitive architecture of chess players is slightly reorganized into functionally and anatomically coherent modules reflecting a distinction between visual, verbal, and executive/attention/processing speed-related functions, perhaps reminiscent of right hemisphere and left hemisphere subnetworks orchestrated by the frontal lobe and its white matter connections.

## 1 Introduction

Chess is a complex intellectual activity that involves multiple cognitive processes (Chase and Simon, 1973; Holding, 1992; Gobet and Simon, 1996; Gobet, 1998; Gobet and Charness, 2006; Bilalić et al., 2010). Chess players encode, remember, and process chess moves through symbolic descriptions, and organize their book knowledge of opening, middlegame themes, and endgame techniques around verbal labels (Holding, 1992). It has been suggested that intense chess practice may have a positive impact on cognitive performance. For example, Elo (1978) found that top chess players had superior verbal skills, represented by a high frequency of professional writing occupations and mastery of foreign languages. Chess practice may also impact perceptual processing. For example, expert chess players recognize game plays and recall positions thanks to their perceptual organization and internal representation of positions (Chase and Simon, 1973; Gobet and Simon, 1998). This enables chess players to make better decisions, as they focus their attention on clusters of pieces forming key game positions rather than fixating on individual pieces, as less experienced players do (Charness et al., 2001).

When it comes to cognitive performance in specific functions, several studies have compared chess players versus non-players across several cognitive measures, using univariate analysis. Some studies showed that chess players seem to have a higher performance in verbal auditory memory (Fattahi et al., 2015), visuospatial working memory (Bachmann and Oit, 1992; Unterrainer et al., 2006), and planning (Unterrainer et al., 2006). However, some studies have found no differences (Unterrainer et al., 2011; Nejati and Nejati, 2012; Hänggi et al., 2014) or even worse performance in chess players compared with non-players in some cognitive measures (Boggan et al., 2012; Nejati and Nejati, 2012). These inconsistencies could be explained by the reduced number of cognitive measures included in most of previous studies, failing to map all cognitive domains and sub-domains of human cognition. Another explanation could be the use of univariate analysis that cannot capture the complexity of intellectual activities such as chess, which engages multiple cognitive domains interactively. Opposite to univariate analysis, multivariate analyses such as graph theory enable the study of complex associations between multiple cognitive measures.

Recent studies have applied graph theory analysis on cognitive measures providing a full characterization of the so-called “cognitive connectome” (Garcia-Cabello et al., 2021), which comprehensively represents the complex organization and associations among multiple cognitive measures in a population. Using graph theory analysis on cognitive measures provides rich data on the centrality of specific cognitive measures in the connectome as well as information on the integration and segregation of the cognitive connectome (Tosi et al., 2020). However, no previous study has used multivariate analysis or investigated the cognitive connectome of chess players. Such a study could help clarify the presumable advantage of intensive intellectual practice of activities such as chess, informing on the potential benefits of chess for brain stimulation or risk reduction of age-related cognitive decline. A graph theory approach may likely better reflect the multivariate and integrated way the human brain faces a complex cognitive demand such as playing chess.

The overall goal of the current study was to investigate the cognitive connectome of adult chess players. Firstly, we performed “baseline” univariate analysis to be able to compare with the results obtained from graph theory in subsequent aims. This univariate analysis consisted of a comparison of cognitive performance of chess players versus controls across cognitive measures. Secondly, we performed multivariate analyses (graph theory) by comparing chess players and controls across global and nodal graph measures of integration, segregation, and centrality. Thirdly, we performed a separate analysis of community structures in chess players and controls, to gain additional understanding of the cognitive architecture of chess players. We hypothesized that chess player would perform better than controls in cognitive functions previously associated to chess practice (executive functions/planning, attention, precession speed, and memory), but we anticipated that graph theory analyses would detect more differences between groups than univariate analysis. In addition, we expected that chess players would have a more efficient and integrated cognitive connectome as directly reflected by the graph measures but also through a lower number of modules in the analysis of community structures.

## 2 Methods

### 2.1 Participants

We recruited a total of 38 participants (19 chess players and 19 controls) with ages between 39 and 69 years. The chess group was recruited through announcements at the chess federation and several chess clubs in Tenerife (Spain). The control group was selected from the GENIC-database (Group of Neuropsychological Studies of the Canary Islands) (Machado et al., 2018) by matching controls to chess players on age, sex, education level, and manual preference using the Edinburgh Handedness Inventory (Oldfield, 1971; Table 1). All participants were male by study design. Both study groups reported leisure and physical activities, making sure that none of the controls played chess regularly or fulfilled the inclusion criteria of the chess group. Furthermore, we assessed crystallized intelligence with the Wechsler Adult Intelligence Scale (WAIS-III) Information subtest (Wechsler, 1997a).

**Table 1:** Demographic characteristics.

	Chess players (*n* = 19)	Controls (*n* = 19)	Estimate	*p-*value
Age, years (min – max)	49.5 (7.8) (39–69)	49.7 (7.7) (39–69)	173.5	0.837
Sex (Male, n)	19	19	–	–
Education level			–	–
Completed secondary studies	4	4		
University studies	15	15		
WAIS-III Information (min-max)	22.6 (3.6) (16–28)	21.5 (3.4) (14–26)	218	0.270
MMSE (min-max)	29.5 (0.9) (27–30)	28.9 (1.1) (27–30)	237.5	0.062
Elo (min-max)	1733.8 (254.8) (1283–2102)	–	–	–

Estimates are from Mann–Whitney *U* tests. Mean (standard deviation), range from minimum to maximum value for continuous variables, count for categorical variables. Two chess players did not have an Elo rating because they were not actively competing at the time of sample recruitment but they reported to have been playing chess regularly at least 1 h per week for up to 20 years. WAIS-III, Wechsler Adult Intelligence Scale – version III; MMSE, mini-mental state examination.

Participants were assessed with a comprehensive neuropsychological protocol applied by an experienced neuropsychologist. Inclusion criteria for the current study were: (1) normal cognitive performance in comprehensive neuropsychological assessment using age-, sex-, and education-adjusted normative data (2) no neurologic, psychiatric, or systemic diseases with a potential impact on cognitive performance; and (3) no history of substance abuse. Hence, all individuals in this study were within the range of normal cognitive performance. The current study was approved by the ethics committee of the University of La Laguna (Spain), project number 57, and all participants gave their written informed consent, in accordance with The Code of Ethics of the World Medical Association (Declaration of Helsinki).

### 2.2 Neuropsychological assessment

The neuropsychological protocol includes multiple tests of language, processing speed, attention, executive functions, verbal and visual episodic memory, procedural memory, and visuoconstructive, visuoperceptive and visuospatial functions (Supplementary Table S1). In addition, the MMSE (Folstein et al., 1975) was used as a measure of global cognition.

### 2.3 Network construction and graph analysis

**Figure 1:**
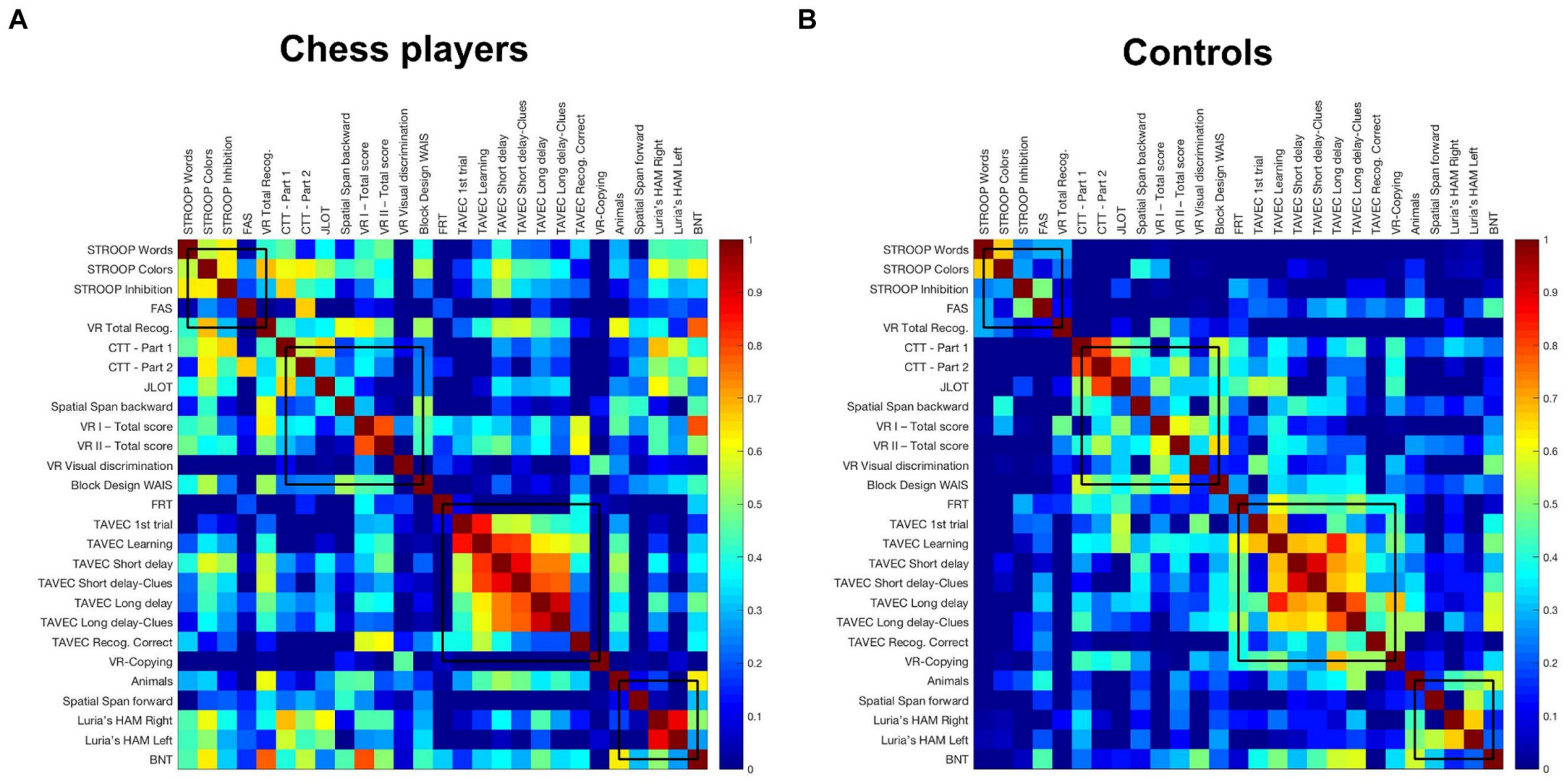
Weighted correlation matrices by study group. Pearson correlation coefficients were used to construct the matrixes. Colder colors represent weaker correlations while warmer colors represent stronger correlations, in a scale from 0 to 1. **(A)** Weighted correlation matrix of chess players; **(B)** weighted correlation matrix of controls.

Table 2 lists the cognitive variables used as nodes for network construction. No cognitive variables from the extensive neuropsychological protocol were excluded for the graph analysis. Prior network construction, variables related to processing speed (i.e., CTT part 1 and part 2) were inverted so that higher scores reflect better performance in all the variables included in the network. The edges between the nodes were calculated through group-specific association matrices of Pearson correlation coefficients from each pair of nodes (Figure 1).

**Table 2:** List of nodes (graph analysis), neuropsychological tests, and cognitive components.

Nodes	Neuropsychological test	Cognitive component
STROOP words	Stroop Test (Golden, 1978)	Sheet 1 Words: processing speed
STROOP colors		Sheet 2 Colors: processing speed
STROOP inhibition		Sheet 3 Inhibition: executive function
CTT - Part 1	Color Trails Test - Part 1 (CTT-1) (D’Elia and Saltz, 1989)	Focusing/visual tracking
CTT - Part 2	Color Trail Test - Part 2 (CTT-2) (D’Elia and Saltz, 1989)	Mental flexibility/executive control
Phonemic fluency (FAS)	Phonemic fluency – FAS (COWAT) (Benton et al., 1989)	Phonemic fluency/executive function
Semantic fluency (animals)	Semantic fluency – animals (Benton et al., 1989)	Semantic fluency/executive function
FRT	Facial Recognition Test (FRT-brief version) (Benton et al., 1983)	Visuoperceptive abilities
JLOT - Second half	Judgment of Line Orientation Test (JLOT, H form) (Benton et al., 1983)	Visuospatial abilities
Spatial Span forward	Visuospatial Span – forward and backwards (WMS-III) (Wechsler, 1997b)	Working memory: amplitude
Spatial Span backward		Working memory: manipulation
TAVEC 1st trial	Test de Aprendizaje Verbal España-Complutense (TAVEC, Spanish version of the California Verbal Learning Test (CVLT)) (Benedet and Alejandre, 1998)	Immediate recall (verbal)
TAVEC learning		Immediate recall (verbal)
TAVEC short delay		delayed recall (verbal)
TAVEC short delay-clues		delayed recall (verbal)
TAVEC long delay		delayed recall (verbal)
TAVEC long delay-Clues		delayed recall (verbal)
TAVEC Recog. Correct		recognition subtests (verbal)
VR I – total score	Visual Reproduction Test, (VRT, WMS-III) (Wechsler, 1997b)	Immediate recall (visual)
VR II – total score		Delayed recall (visual)
VR-copying		2-D visuoconstructive abilities
VR total Recog.		Recognition subtests (visual)
VR visual discrimination		Visuoperceptive abilities
Luria’s HAM right	Luria’s Premotor Functions (Luria’s) (Christensen, 1979)	hand alternative movements
Luria’s HAM left		hand alternative movements
Block design WAIS	Block Design – standard and extended version (WAIS-III) (Wechsler, 1997a)	3-D visuoconstructive abilities
BNT	Boston Naming Test (BNT) (Kaplan et al., 1983)	Lexical access by visual confrontation

The resulting correlation matrices were binarized by thresholding correlation coefficients at a range of network densities (min = 10% to max = 45%, in steps of 1%), to ensure fully connected networks and exclude densities where connectomes had random topologies (small-worldness indexes approximating a value of 1). Network topologies of chess players and controls were compared across this range of densities. Following previous studies of cognitive connectomes (Garcia-Cabello et al., 2021), both self-connections and negative correlations were excluded from correlation matrices.

After constructing the networks, we calculated nodal and global graph theory measures. Nodal measures refer to each specific node whereas global measures refer to the average across all nodes. Regarding global measures, we included: the *average global efficiency*, *average local efficiency,* and *transitivity* measures calculated from the binary networks across the different densities, and the *average strength* calculated from the weighted network (before binarization) (Rubinov and Sporns, 2010). The *average global efficiency* is the average inverse shortest path length between a node and the rest of the network, which in contrast to the characteristic path length can be meaningfully computed on disconnected networks (Rubinov and Sporns, 2010). The average global efficiency measures how efficiently information is exchanged throughout the network (Latora and Marchiori, 2001). The mathematical definition of the *average local efficiency* is similar to that of the average global efficiency, but the average local efficiency is restricted to a given node and the subgraph created by the node’s neighbors (Bullmore and Sporns, 2009). The *transitivity* refers to the fraction of a node’s neighbors that are also neighbors of each other in the whole network, normalized by the whole network. It reflects how well the nodes are connected to nearby nodes forming cliques. In a cognitive network, the transitivity reflects whether the cognitive data tend to be organized into communities of cognitive measures that are strongly correlated to nearby cognitive measures, but weakly correlated to cognitive measures belonging to other communities. The *average strength* is given by the sum of the weights of all edges connected to a node. In a cognitive network, the average strength represents the overall magnitude of correlations among cognitive measures in the network (Rubinov and Sporns, 2010).

In addition, we calculated the following nodal measures: *nodal global efficiency*, *nodal local efficiency* (Latora and Marchiori, 2001), and *nodal closeness centrality* (Rubinov and Sporns, 2010). The *nodal global efficiency* of a specific node is the average inverse shortest path length between that node and the rest of the network. The *nodal local efficiency* is the global efficiency of a node calculated on the subgraph created by the node’s neighbors. The *nodal closeness centrality* is similar to the nodal global efficiency but instead of the average is just the inverse of the path length of a node, thus reflecting how central in the connectome a node is.

Finally, to investigate the topology and architecture of the cognitive connectome of chess players, we also performed modular analyses by applying the Louvain algorithm (Blondel et al., 2008) on weighted undirected networks with a gamma value of 1.

The formulae used to calculate all these graph measures are provided in Rubinov and Sporns (2010) and Latora and Marchiori (2001).

### 2.4 Statistical analysis

Analyses were performed using the R programming environment (R Core Team, 2016) and BRAPH^1^ (Mijalkov et al., 2017).

All variables were assessed to confirm the absence of outliers. The distribution of the data was ensured to follow the normal distribution by inspecting histograms and computing indexes of asymmetry and kurtosis. For demographic variables, we used Mann–Whitney *U* tests as these variables did not follow the normal distribution.

We addressed our first study aim by comparing chess players versus controls across 27 cognitive variables, using univariate analyses consisting of *t*-tests for continuous variables and Mann–Whitney *U* tests for continuous variables that did not conform to a normal distribution. To assess effect sizes, Cohen’s was used for independent samples *t*-tests and Hedge’s g was used for Mann–Whitney *U* tests. We confirmed that variances were homogenous. In all these analyses, significant differences were considered when *p* ≤ 0.05 (two-tailed). Our second study aim was addressed by comparing global and nodal graph measures across groups. Between-group comparisons of global graph measures were conducted through 1,000 nonparametric permutations over the previously pre-specified range of network densities (min = 10% to max = 45%, in steps of 1%). The 95% confidence intervals of each distribution were used as critical values for testing the null hypothesis at *p* ≤ 0.05 (two-tailed). Nodal graph analyses were also performed over the 10–45% range of network densities, and results were reported at the median density (27.5%), as in previous studies (Ferreira et al., 2019; Garcia-Cabello et al., 2021). Nonetheless, we ensured that results at the median density were representative of neighboring densities, to focus on stable differences across the range of densities. *p*-values in nodal analysis were adjusted with the false discovery rate (FDR) method for multiple testing. We addressed our third study aim by inspecting differences in community structure between chess players and controls.

## 3 Results

Table 1 shows the demographic characteristics of chess players and controls. Both groups were statistically comparable in terms of crystallized intelligence as measured by the WAIS-III Information subtest.

### 3.1 First aim: univariate analysis across cognitive measures

To address the first aim of this study, we compared the cognitive performance of chess players versus controls across 27 cognitive variables (Table 3). Chess players outperformed the control group in JLOT – Second half (*p* = 0.039), with a medium effect size (*g* = 0.75). We did not observe any statistically significant difference in any other cognitive measure. However, visual inspection of effect sizes showed moderate effects (g/d > 0.5) in STROOP Inhibition (*d* = 0.56), semantic fluency (animals) (*d* = −0.55), spatial span forward (*g* = 0.56), and visual discrimination VR (*g* = 0.55).

**Table 3:** Cognitive performance: results from univariate analysis.

	Chess players (*n* = 19)		Controls (*n* = 19)		Estimate	*p-*value	*Effect s*ize
Variables	*M*	SD	*M*	SD			
STROOP words	106.6	14.5	106.8	12.6	−0.059	0.953	−0.02
STROOP colors	76.6	9.3	73.7	8.4	1.018	0.315	0.34
STROOP inhibition	48.3	6.9	44.1	8.4	1.690	0.099	0.56
CTT – Part 1	34.7	11.2	37.7	17.2	173.5	0.837	−0.20
CTT – Part 2	84.1	20.9	85.7	31.8	203	0.511	−0.05
Phonemic fluency (FAS)	44.9	10.0	44.8	11.4	0.030	0.976	0.01
Semantic fluency (animals)	23.1	4.2	26.0	6.1	−1.658	0.105	−0.55
FRT	23.4	2.0	22.8	1.9	0.896	0.375	0.30
JLOT – second half	13.1	1.9	11.4	2.5	250	**0.039**	0.75
Spatial span forward	9.7	1.3	8.8	1.8	233.5	0.112	0.56
Spatial span backward	9.2	1.7	8.7	1.7	213	0.333	0.28
TAVEC 1st trial	6.4	2.3	6.7	2.3	173	0.824	−0.12
TAVEC learning	56.0	9.3	55.7	8.7	0.089	0.929	0.03
TAVEC short delay	12.5	2.8	12.3	2.8	187.5	0.836	0.07
TAVEC short delay-clues	12.5	2.8	13.6	2.1	141.5	0.249	−0.43
TAVEC long delay	14.6	1.8	14.0	2.4	206	0.429	0.27
TAVEC long delay-clues	14.8	1.6	14.6	1.8	198.5	0.576	0.11
TAVEC recog. correct	15.7	0.4	15.5	0.6	211	0.259	0.38
VR I – total score	87.3	12.3	86.8	9.2	0.134	0.894	0.04
VR II – total score	79.1	19.0	78.4	16.1	188.5	0.815	0.03
VR copying	99.8	2.7	100.0	3.5	170	0.757	−0.06
VR total recognition	45.0	2.1	45.3	2.0	162.5	0.594	−0.14
VR visual discrimination	6.8	0.3	6.6	0.4	218.5	0.145	0.55
Luria’s HAM right	21.7	7.6	18.5	4.7	222.5	0.218	0.49
Luria’s HAM left	21.3	6.5	20.5	5.1	0.412	0.682	0.14
Block design WAIS	44.7	10.2	45.3	7.5	−0.198	0.843	−0.07
BNT	28.6	1.4	28.6	1.6	174.5	0.856	0

Estimates are from independent samples *t*-tests for normally distributed variables and Mann–Whitney *U* tests for non-parametric distributed variables. Student’s *t*-test was used for Stroop Words, Stroop Colors, Stroop Inhibition, Phonemic Fluency (FAS), Semantic Fluency (animals), Facial Recognition Test (FRT), TAVEC Learning, Total score - VR I, left-hand alternation (Luria’s HAM Left), and Block Design (WAIS). For the remaining variables, the Mann–Whitney *U* test was used. Cohen’s *d* or Hedges’s *g* for effect sizes. Significant differences = *p* < 0.05 highlighted in bold. M, mean; SD, standard deviation; CTT, color trail test; FRT, facial recognition test; JLOT, judgment of line orientation test; VR, visual reproduction (parts I and II); Luria’s HAM, hand alternating movements; BNT, Boston naming test.

### 3.2 Second aim: graph analysis on the cognitive connectome

The weighted correlation matrices of the cognitive connectomes of chess players and controls are displayed in Figure 1, with variables sorted out by modules according to the modular analysis of community structures of controls (see section 3.3. below). Visual inspection of the matrices showed that chess players had a more widespread pattern of correlations than controls. In particular, executive measures tended to correlate strongly with themselves and with measures of premotor function, visuospatial ability, visuoconstructive ability, visual memory, and naming, forming a cluster of between-module correlations that was not as salient in controls. In addition, verbal memory measures tended to correlate strongly with themselves and with some measures of executive function as well as with semantic fluency and visual memory recognition, which was not as salient in controls. Therefore, there were numerous strong correlations in chess players both between-modules and within-module, suggesting greater integration in chess players. In contrast, controls tended to have less widespread correlations with less correlations between-modules than chess players.

**Figure 2:**
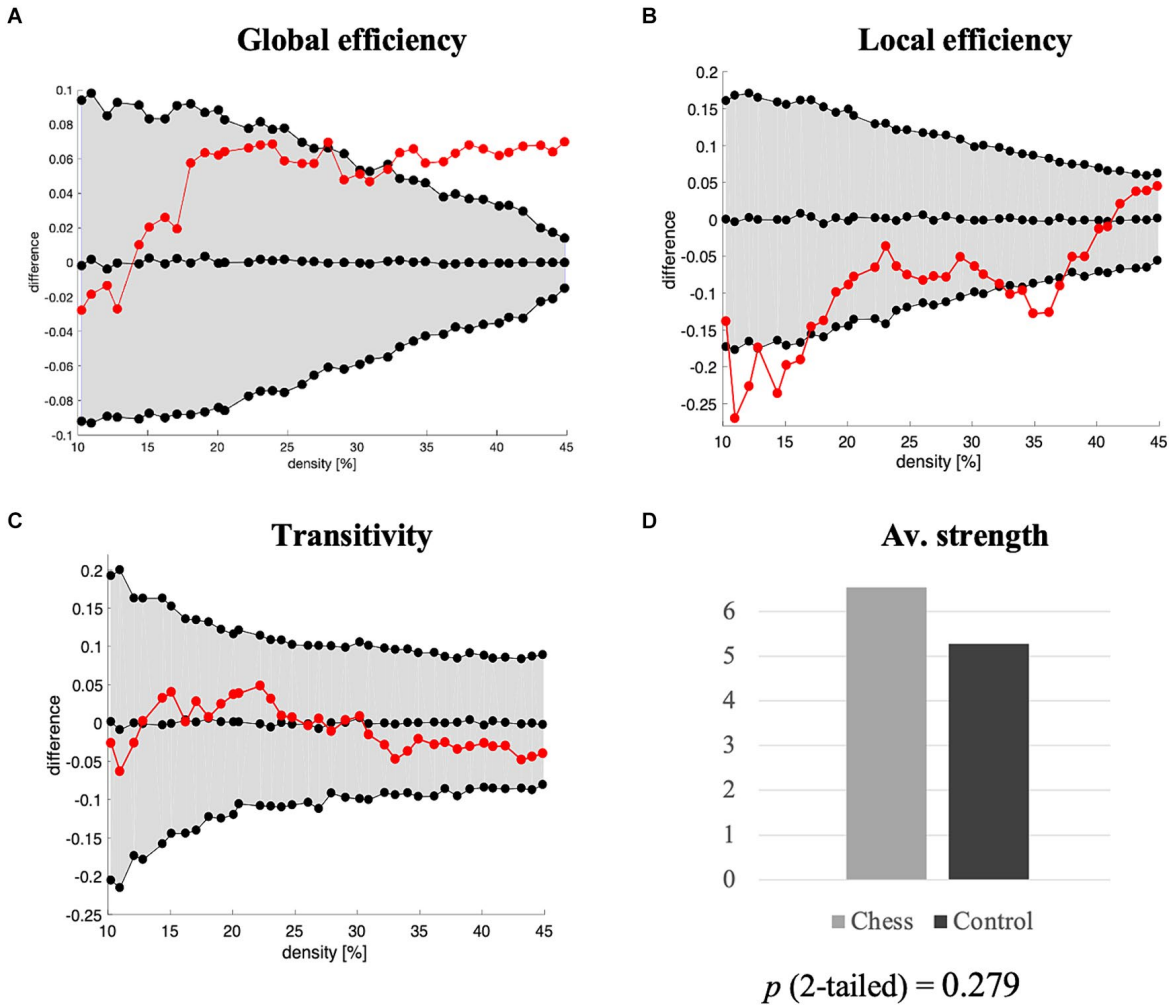
Global graph measures. The figure illustrates global graph measures of **(A)** global efficiency, **(B)** local efficiency, and **(C)** transitivity, with network densities on the *x*-axis ranging from min = 10% to max = 45%, in steps of 1%. Group differences are depicted on the *y*-axis. Differences are significant when red circles fall outside the shaded area in gray color (95% confidence intervals), with controls as the depicted group (positive differences mean higher values in controls). **(D)** Average strength was calculated on weighted networks.

The quantitative analysis of global graph measures showed that chess players had a higher average local efficiency and a lower average global efficiency, compared with the control group (Figure 2). There were no statistically significant group differences in transitivity or average strength. Hence, chess players had greater integration at the local level, but not at the global level, with o differences in segregation.

In the nodal graph analysis, chess players showed a higher nodal closeness centrality in STROOP Words, STROOP Colors, STROOP inhibition, VR Copying, VR Total Recognition, and Luria’s hand alternating movements (HAM) with the right hand, compared with the control group. Conversely, the control group showed a higher nodal closeness centrality in FAS. There were no statistically significant differences between groups in nodal global efficiency or nodal local efficiency (Table 4).

**Table 4:** Nodal graph analysis.

Graph measure and cognitive variable	Chess players	Controls	FDR-adjusted *p*-value
Nodal global efficiency			*n.s.*
Nodal local efficiency			*n.s.*
Closeness centrality			
STROOP words	0.6216	0.2708	<0.05
STROOP colors	0.7419	0.3662	<0.05
STROOP inhibition	0.5750	0.3210	<0.05
FAS	0.3485	0.4643	<0.05
VR copying	1	0.5417	<0.05
VR total recognition	0.6970	0.3171	<0.05
VR visual discrimination	1	0.5098	<0.05

Nodal analyses were performed on all the variables listed in Table 3, while only significant nodes are displayed in Table 4. VR I, visual Reproduction (part I); FAS, phonemic fluency; FDR, false discovery rate.

### 3.3 Third aim: architecture of cognitive connectome through modular analysis of community structures

**Figure 3:**
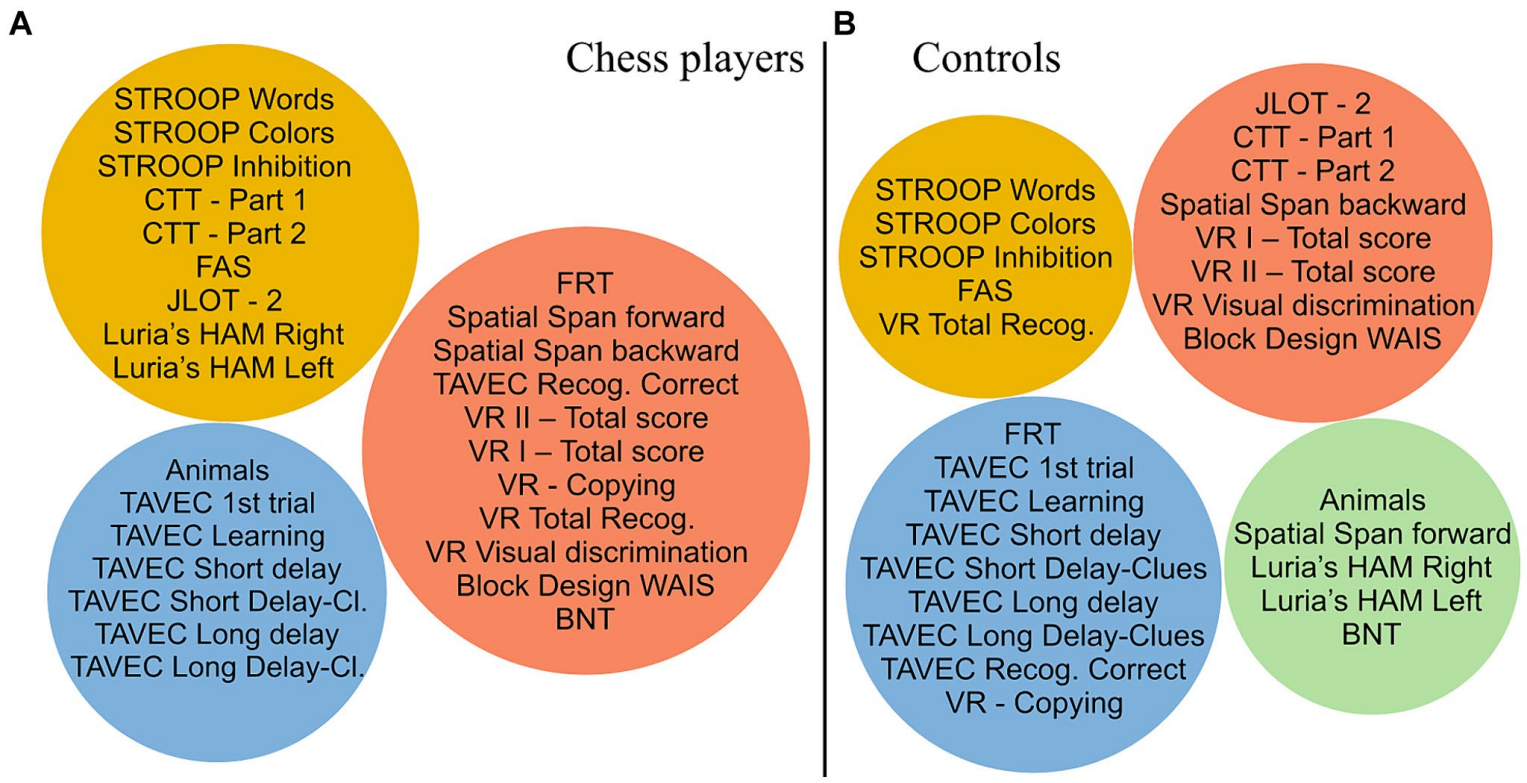
Architecture of cognitive connectomes from modular analysis of community structures. After modular analysis, each resulting module was represented by a different color (blue, orange, yellow, and green) for both study groups. **(A)** Cognitive architecture of the chess players’ connectome and **(B)** Cognitive architecture of the control group’s connectome.

Thirdly, we analyzed differences in community structure between chess players and controls. This analysis revealed distinct modular topologies in chess players and controls (Figure 3). Chess players showed three modules while controls showed four modules. In addition, the cognitive architecture of chess players was organized more coherently with modules including functionally/anatomically similar cognitive functions as follows: an executive/processing speed module (yellow circle in Figure 3), a visual module including visual memory, visuoconstructive, visuoperceptive, and visuospatial measures (orange circle), and a verbal memory module (blue circle). However, in the control group, the modules included somehow mixed cognitive functions, with a less coherent architecture, as follows: an executive/processing speed module (yellow circle in Figure 3), a module including executive, visual memory, and visuospatial measures (orange circle), a module including verbal memory, visuoconstructive, and visuoperceptive measures (blue circle), and a module including premotor, semantic, and visuospatial measures (green circle).

## 4 Discussion

The overall goal of the current study was to investigate the cognitive connectome of adult chess players. We found that chess players had a more integrated connectome locally, where nodes related to executive/attention/processing speed and visuoconstructive abilities played a central role. Furthermore, chess players had a slightly reorganized cognitive architecture into three functionally and anatomically coherent modules, in the context of better cognitive performance in visuospatial abilities and large effect sizes of differences in executive/attention/processing speed, semantic fluency, and visual abilities, when compared with controls.

The univariate analysis demonstrated that chess players perform better than controls in visuospatial abilities. High performance in visuospatial abilities in chess players was also reported in a previous study (Saariluoma et al., 2004). In addition, previous studies reported differences in other cognitive functions such as memory, attention, verbal skills, processing speed, and executive functions (Chase and Simon, 1973; Bachmann and Oit, 1992; Gobet and Simon, 1996; Reingold et al., 2001; Unterrainer et al., 2006; Bilalić et al., 2010, 2012; Fattahi et al., 2015; Burgoyne et al., 2016). While we did not find any statistically significant differences beyond visuospatial abilities, we did observe large effect sizes of differences with chess players performing better in executive/attention/processing speed and visual abilities, in line with the cited studies. Therefore, it is possible that those differences also existed in our cohort but were difficult to capture due to our slightly smaller sample size than in Bachmann and Oit (1992), Fattahi et al. (2015), and Unterrainer et al. (2006); or because most previous studies included younger samples, while group differences could be mitigated with increasing age. An interesting prospect for future studies would be to investigate whether the positive effect of chess on cognition diminishes with increasing age.

We expanded the previous research based on univariate analyses by performing graph theory analysis on cognitive measures. Visual inspection of the cognitive connectomes revealed that chess players had a more widespread pattern of correlations. In particular, executive/attention measures correlated strongly with themselves and with measures of premotor function, visuospatial ability, visuoconstructive ability, visual memory, and naming. In addition, verbal memory measures correlated strongly with themselves and with measures of executive function/attention, semantic fluency, and visual memory recognition. This translates into a pattern of increased between- and within-module connectivity in chess players, perhaps explained by the high cognitive demand of intensive chess practice over time. Modules would reflect cognitive (but possibly, also functional or structural) subnetworks. This means that chess players may have an advantage to integrate different subnetworks or distribute information rapidly across subnetworks. Previous univariate studies highlighted the proficiency of chess players in tests of executive function/attention, memory, and visual ability (Bachmann and Oit, 1992; Reingold et al., 2001; Unterrainer et al., 2006; Bilalić et al., 2010, 2012; Fattahi et al., 2015; Burgoyne et al., 2016).

The modular analysis of community structures further supports the idea of a potential reorganization of the cognitive connectome in chess players. We observed that chess players had less modules than controls, with a more functionally and anatomically coherent architecture. In particular, we observed a first module including executive/attention/processing speed abilities, a second module including visual abilities, and a third module including verbal abilities. This architecture suggests a frank separation between traditionally considered right hemisphere (second module) and left hemisphere (third module) abilities, with the additional role of the frontal lobe and its white matter connectivity (first module) to possibly orchestrate the full connectome. This architecture contrasts with the less coherent and more globally integrated organization of the control group.

We further substantiated our qualitative inspection of connectomes and analysis of community structures by performing quantitative analyses on global and nodal graph theory measures. Indeed, we found that chess players had a higher average local efficiency than controls. A previous study reported differences between expert chess players and novice players using graph theory analysis on resting-state functional magnetic resonance imaging (rs-fMRI) (Duan et al., 2014). They observed that grandmaster and master level chess players had a higher clustering coefficient than novice players. That finding partly aligns with our result on average local efficiency. While the average local efficiency primary is a measure of integration of short connections, it can also be interpreted as a measure of segregation, like the clustering coefficient: i.e. strong short connections (high local efficiency) together with weak long connections (low global efficiency) define a more segregated network, which would be signified by a higher clustering coefficient. Hence, our study expands the previous rs-fMRI findings of Duan et al. (2014) by demonstrating that the advantageous functional connectivity in chess players seems to permeate the cognitive connectome, thus preliminarily linking brain and behavioral results.

We also observed that chess players had a lower average global efficiency than controls. Global efficiency reflects whether nodes correlate with each other forming short paths, with higher global efficiency values reflecting the capacity to quickly distribute information across the connectome via short paths (Sporns, 2018). In the context of our study, the average global efficiency reflects how the performance in a specific cognitive task contributes to performance in other tasks within the connectome (between-module or subnetwork connectivity). In contrast, the average local efficiency is restricted to a given node and the subgraph created by other nodes belonging to the same community (within-module or subnetwork connectivity). It is possible that the low global efficiency in chess players is a consequence of the high local efficiency, without affecting the segregation of their connectome. The intensive training of a visual subnetwork and the high demands on an executive/attention/processing speed subnetwork as informed by our analysis of community structures may rewire the connectome of chess players into a highly specialized connectome to face the demands of a task like chess. This would be at the cost of a lower capacity to integrate the connectome globally, or at least not in the same balanced way controls integrate their connectome globally, because perhaps the verbal subnetwork is not as intensively trained as the visual subnetwork in chess players. Our finding of chess players having a lower nodal closeness centrality in verbal fluency (FAS) would support this idea. This interpretation of global efficiency remains speculative at the moment but is supported by several previous studies where we demonstrated a similar pattern of results with a lower global efficiency in people performing high in a linguistic task, presumably because of their greater capacity to recruit non-linguistic cognitive networks (between-subnetwork connectivity), and an efficient use of language networks (within-subnetwork connectivity) (Gonzalez-Burgos et al., 2020; Gonzalez-Burgos et al., 2021; Mohanty et al., 2021). This hypothesis would be further supported by rs-fMRI, electroencephalography (EEG), or single photon emission computerized tomography (SPECT) studies that showed an increased brain activation in posterior brain regions, the non-dominant hemisphere, and subcortical structures in response to a high cognitive demand or difficulty level (Onofrj et al., 1995; Duan et al., 2012; Villafaina et al., 2019, 2021; Song et al., 2020). Hence, we suggest that the high local efficiency in the cognitive connectome of chess players may be at the cost of a low global efficiency, possibly resulting in a higher specialized performance. This more integrated connectome locally at the cost of a less integrated connectome globally may be due to the central role of executive/attentional/processing speed and visuoconstructive nodes in the connectome of chess players, as reflected by our nodal analyses.

We cannot discuss our graph theory results with similar studies in chess players because of the lack of such studies. Nonetheless, two previous studies used graph theory on cognitive data from cognitively unimpaired individuals (Garcia-Cabello et al., 2021; Wright et al., 2021), like the population in our study. They both demonstrated that the cognitive connectome exhibits changes across age that likely mitigate the effects of aging on cognitive performance (Garcia-Cabello et al., 2021; Wright et al., 2021). In a recent study, we investigated the effect of cognitive reserve on the cognitive connectome, as well as how cognitive reserve may modulate the cognitive connectome across age groups (Habich et al., 2024). We found that individuals with a high cognitive reserve exhibited a more stable cognitive connectome across ages in terms of segregation and integration, compared to individuals with a low cognitive reserve. This pattern was associated with a better cognitive performance in individuals with a high cognitive reserve across all age groups (Habich et al., 2024). Hence, cognitive reserve and activities that are highly stimulating such as playing chess, may influence the cognitive connectome in rather similar ways. This influence may translate into certain cognitive benefits. For chess, these benefits may include specific capabilities for decision-making, problem-solving, attentional processes, working memory, and perceptual processes—cognitive functions that are activated during a chess game.

Overall, we demonstrate the capacity of graph theory to identify differences in the cognitive connectome of chess player, above and beyond the differences detected by univariate analyses. Nonetheless, we acknowledge some limitations in our study. Firstly, by design, we restricted our cohort to men due to the low representation of women chess players in the sources we recruited our participants. The lower representation of women chess players is common in previous studies. The reason for this underrepresentation is unknown, but some authors have suggested that it may be related to social factors such as the lack of female chess role models or because different gender socialization experiences may lead to different interests or motivations for free time activities (Charness and Gerchak, 1996; Bilalić and McLeod, 2006; Chabris and Glickman, 2007; Bilalić et al., 2009; Blanch et al., 2015). Although we are not aware of previous studies investigating sex differences in the cognitive connectome, it would be interesting to test whether the cognitive connectome of men and women chess players differ. Secondly, the sample size of our study is relatively small, although it is similar or exceeds the sample size of some previous chess studies (Brockmole et al., 2008; Bilalić et al., 2009; Kiesel et al., 2009; Bilalić et al., 2010, 2012). This issue of small sample sizes in chess studies in general may be due to difficulties in identifying and recruiting the study population. Nonetheless, despite the current sample size, we could fulfil the goal of this study of comparing graph theory results with univariate analysis results. Thirdly, we limited our analyses to compare chess players with controls, while the degree of practice, expertise level, and other variables related to chess proficiency could have an additional impact on the cognitive connectome and architecture of chess players. Fourthly, we did not perform any corrections for multiple comparisons in univariate analysis, while *p*-values in nodal graph analyses were adjusted using the FDR method. Despite this conservative choice for the graph theoretical analyses, we could validate our hypothesis that graph analysis would reveal more significant differences than univariate analysis. Fifthly, although we did not account for the potential influence of group differences in leisure and physical activities or diet, we did ensure that both groups were comparable in crystallized intelligence. In our cohort, crystallized intelligence reflects the individual’s interests (Correia et al., 2015) and correlates with a questionnaire of cognitive reserve that includes performance of cognitively stimulating activities (Ferreira et al., 2016). Our study thus serves to identify some key characteristics of the cognitive connectome of chess players and, if replicated in independent and larger cohorts, future studies could expand the current findings towards defining the additional impact of chess proficiency on the cognitive connectome. Finally, our current analyses are cross-sectional and we cannot fully demonstrate causal relations between chess practice and cognitive performance.

We conclude that, in our cohort, the connectome of chess players was more integrated locally than that of healthy controls, but at the cost of a less integrated connectome globally. This may be due to the central role of executive/attention/processing speed and visuoconstructive nodes in the connectome of chess players, while the differences do not seem to permeate segregation aspects of their connectome. Based on these findings, we suggest that the cognitive architecture of chess players may be slightly reorganized into functionally and anatomically coherent modules reflecting a distinction between visual, verbal, and executive/attention/processing speed related functions. This architecture could be reminiscent of right hemisphere and left hemisphere subnetworks orchestrated by the frontal lobe and its white matter connections, partly favoring chess players on cognitively demanding tasks with a strong visual component. Future studies should integrate neuroimaging and cognitive data to advance our current understanding on the potential impact of chess on brain functioning and cognitive performance.

## Data Availability

The raw data supporting the conclusions of this article will be made available by the authors upon reasonable request of qualified researchers.

## Footnotes

^1^http://braph.org
