# Supplementary material for: The effect of chess on cognition: a graph theory study on cognitive data
Source: Front Psychol. 2024 Sep 17;15:1407583. doi: 10.3389/fpsyg.2024.1407583 (PMC11442243; doi:10.3389/fpsyg.2024.1407583)
Supplement: Supplementary file 1 [file Table_1.DOCX]

Supplementary Material

# Supplementary Table

**Table S1. List of neuropsychological tests grouped by cognitive functions.**

| **Cognitive functions - Neuropsychological test** |
| --- |
| ***Global cognition and clinical variables*** |
| Mini-Mental State Examination (MMSE) (Folstein, Folstein and McHugh, 1975) |
| Blessed Dementia Rating Scale (BDRS) (Blessed, Tomlinso and Roth, 1968) |
| Functional Activity Questionnaire (FAQ) (Pfeffer *et al.*, 1982) |
| Geriatric Depression Scale, Spanish version (GDS-VE) (Martínez de la Iglesia *et al.*, 2002) |
| Beck’s Depression Inventory (BDI) (Beck *et al.*, 1961) |
| WAIS-III Information subtest (WAIS-III) (Wechsler, 1997a) |
| ***Processing Speed and Attention*** |
| Color Trails Test - Part 1 (CTT-1) (D’Elia and Saltz, 1989) |
| ***Visuospatial, visuoconstructive, and visuoperceptive functions*** |
| Judgment of Line Orientation Test (JLOT, H form) (Benton *et al.*, 1983) |
| Facial Recognition Test (FRT-brief version) (Benton *et al.*, 1983) |
| Block Design – standard and extended version (WAIS-III) (Wechsler, 1997a) |
| Visual Reproduction Test, Copy subtest (VRT, WMS- III) (Wechsler, 1997b) |
| Visual Reproduction Test, Visual Discrimination subtest (VRT, WMS- III) (Wechsler, 1997b) |
| ***Working Memory, Executive Functions, and Premotor Functions*** |
| Color Trail Test - Part 2 (CTT-2) (D’Elia and Saltz, 1989) |
| Visuospatial Span – forward and backwards (WMS-III) (Wechsler, 1997b) |
| Stroop Test (Golden, 1978) |
| Phonemic fluency – FAS (COWAT) (Benton, Hamsher and Sivan, 1989) |
| Semantic fluency – animals (Benton, Hamsher and Sivan, 1989) |
| Luria’s Premotor Functions (Luria’s) (Christensen, 1979) |
| ***Learning and Memory*** |
| *Test de Aprendizaje Verbal España-Complutense* (TAVEC, Spanish version of the California Verbal Learning Test (CVLT)) (Benedet and Alejandre, 1998) |
| Visual Reproduction Test, (VRT, WMS-III) (Wechsler, 1997b) |
| ***Language*** |
| Boston Naming Test (BNT) (Kaplan, Goodglass and Weintraub, 1983) |

**Supplementary references**

Beck, A.T. *et al.* (1961) ‘An inventory for measuring depression.’, *Archives of general psychiatry*, 4, pp. 561–571.

Benedet, M. and Alejandre, M. (1998) *TAVEC: Test de Aprendizaje Verbal España-Complutense. Manual*. Madrid: TEA ediciones.

Benton, A. *et al.* (1983) *Contributions to neuropsychological assessment: A clinical manual.* Edited by O.U. Press. New York.

Benton, A., Hamsher, K. and Sivan, A. (1989) *Multilingual aphasia examination. Iowa City, IA: AJA Associates*. 2nd Ed. Edited by A. Associates. Iowa City, IA: University of Iowa.

Blessed, G., Tomlinso, B.E. and Roth, M. (1968) ‘Association between quantitative measures of dementia and of senile change in cerebral grey matter of elderly subjects’, *BRITISH JOURNAL OF PSYCHIATRY*, 114(512), pp. 797–811. Available at: https://doi.org/10.1192/bjp.114.512.797.

Christensen, A.-L. (1979) *Luria’s neuropsychological investigation*. 2nd Ed. Copenhagen: Munksgaard.

D’Elia, L. and Saltz, P. (1989) *Color Trail 1 and 2*. Odessa, FL: Psychological Assessment Resources.

Folstein, M.F., Folstein, S.E. and McHugh, P.R. (1975) ‘“Mini-mental state”. A practical method for grading the cognitive state of patients for the clinician’, *Journal of Psychiatric Research*, 12(3), pp. 189–198. Available at: https://doi.org/10.1016/0022-3956(75)90026-6.

Golden, C.J. (1978) *Stroop Colour and Word Test: A manual for clinical and experimental uses.* Chicago, Illinois: Stoelting Company.

Kaplan, E.F., Goodglass, H. and Weintraub, S. (1983) *The Boston Naming Test*. 2nd Ed. Philadelphia: Lea & Febiger.

Martínez de la Iglesia, J. *et al.* (2002) ‘Versión española del cuestionario de Yesavage abreviado (GDS) para el despistaje de depresión en mayores de 65 años: adaptación y validación’, *Medifam*, 12(10), pp. 620–630. Available at: https://doi.org/10.4321/S1131-57682002001000003.

Pfeffer, R.I. *et al.* (1982) ‘Measurement of Functional Activities in Older Adults in the Community’, *Journal of Gerontology*, 37(3), pp. 323–329. Available at: https://doi.org/10.1093/geronj/37.3.323.

Wechsler, D. (1997a) *Wechsler Adult Intelligence Scale - Administration and Scoring Manual (3rd ed.)*. San Antonio: The Psychological Corporation.

Wechsler, D. (1997b) *Wechsler Memory Scale – Third Edition Technical Manual*. 3rd ed. San Antonio, Texas: The Psychological Corporation.
